# Supplementary material for: Proteomic Landscape of Human Spermatozoa: Optimized Extraction Method and Application
Source: Cells. 2022 Dec 15;11(24):4064. doi: 10.3390/cells11244064 (PMC9776871; doi:10.3390/cells11244064)
Supplement: Supplementary file 1 [file cells-11-04064-s001.zip › Supplementary figures.pdf]

Supplementary Information

Proteomic Landscape of Human Spermatozoa: Optimized Extraction Method and Application

Mengqi Luo [1,†], Tao Su [1,†], Shisheng Wang [1], Jianhai Chen [1], Tianhai Lin [2], Qingyuan Cheng [3], Younan Chen [1,4,5], Meng Gong [1,4,5], Hao Yang [1,4,5], Fuping Li [3,*] and Yong Zhang [1,4,5*]

[1] Laboratory of Clinical Proteomics and Metabolomics, Institutes for Systems Genetics, Frontiers Science Center for Disease-Related Molecular Network, West China Hospital, Sichuan University, Chengdu 610017, China; mqiluo@126.com (M.L.); s.t.15908@foxmail.com (T.S.); shishengwang@wchscu.cn (S.W.); jianhaichen@scu.edu.cn (J.C.); chenyounan@scu.edu.cn (Y.C.); gongmeng@scu.edu.cn (M.G.); yanghao@scu.edu.cn (H.Y.)

[2] Department of Urology, West China Hospital, Sichuan University, Chengdu 610017, China; tlin@scu.edu.cn (T.L.)

[3] Human Sperm Bank, Key Laboratory of Birth Defects and Related Diseases of Women and Children of Ministry of Education, West China Second University Hospital of Sichuan University, Chengdu 610017, China; cqy791430418@outlook.com (Q.C.)

[4] Organ Transplant Center and NHC Key Lab of Transplant Engineering and Immunology, West China Hospital, Sichuan University, Chengdu 610017, China

[5] Sichuan Provincial Engineering Laboratory of Pathology in Clinical Application, West China Hospital, Sichuan University, Chengdu 610017, China

* Correspondence: lfpsnake@scu.edu.cn (F.L.); nankai1989@foxmail.com (Y.Z.); Tel.: +86-28-85164031; Fax: +86-28-85164031.

† These authors contributed equally to this work.

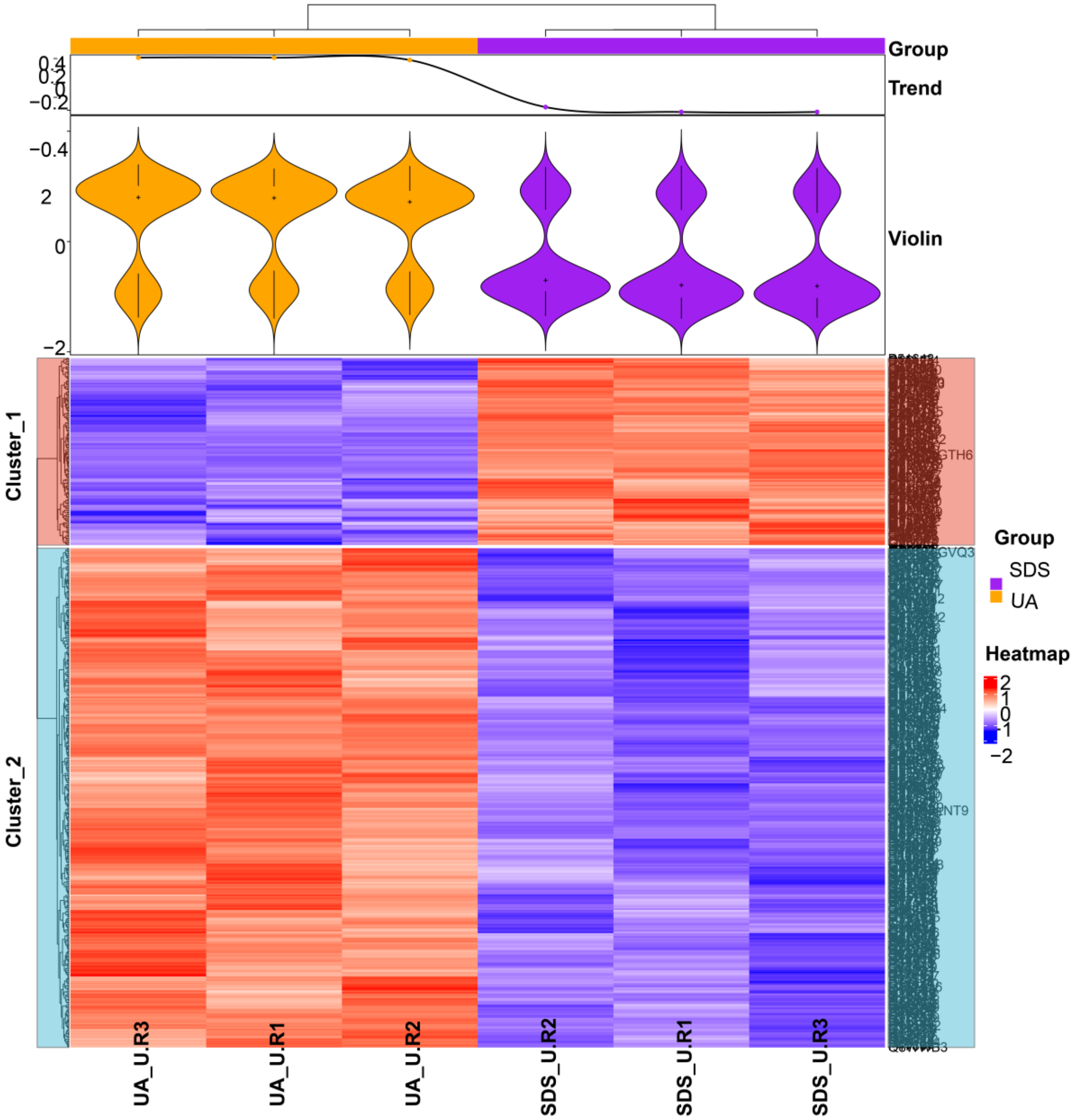

Figure S1. Heatmap analysis of different proteins between UA_Ultrasonication and SDS_Ultrasonication methods.

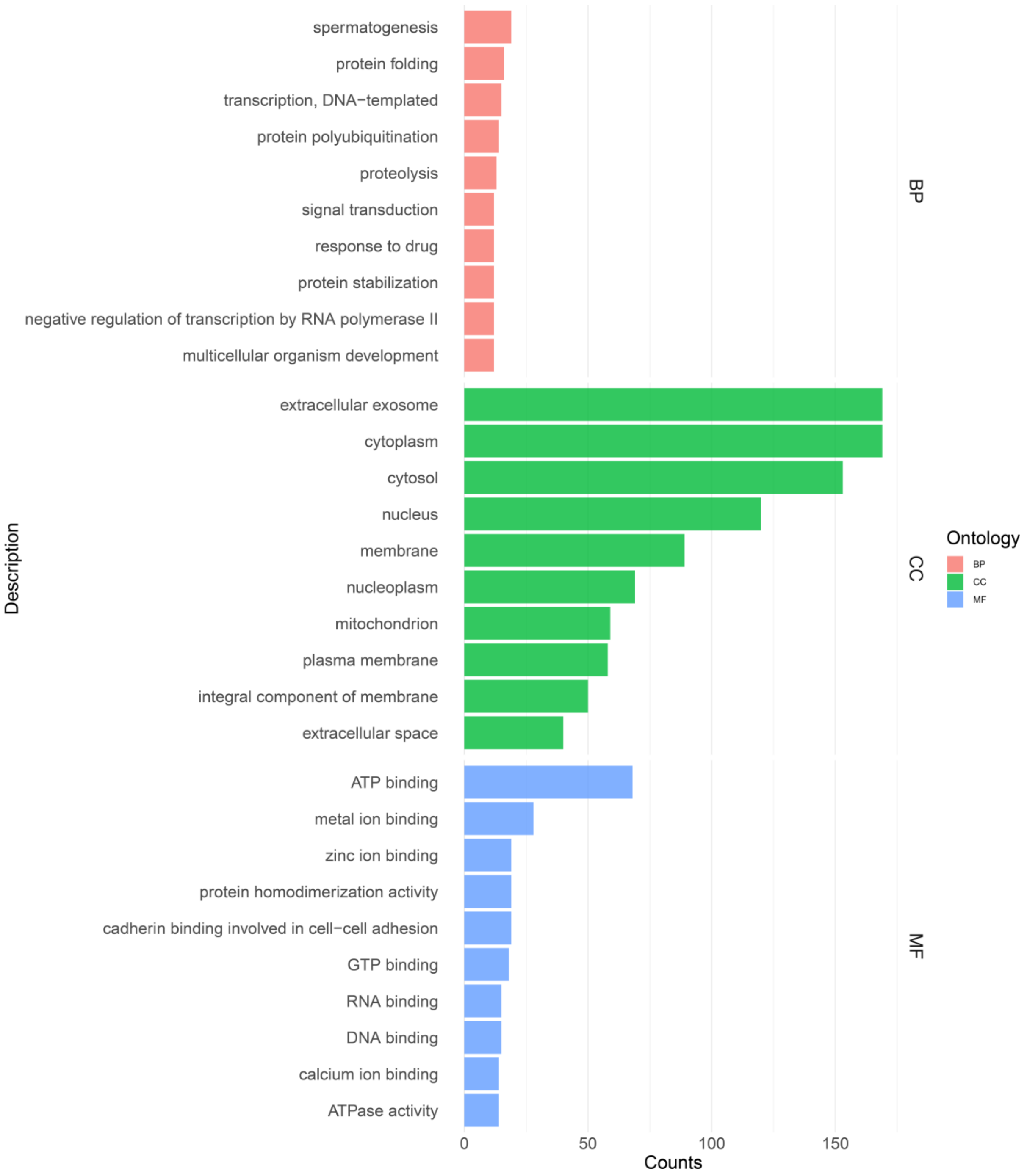

Figure S2. GO analysis of different proteins between UA_Ultrasonication and SDS_Ultrasonication methods.

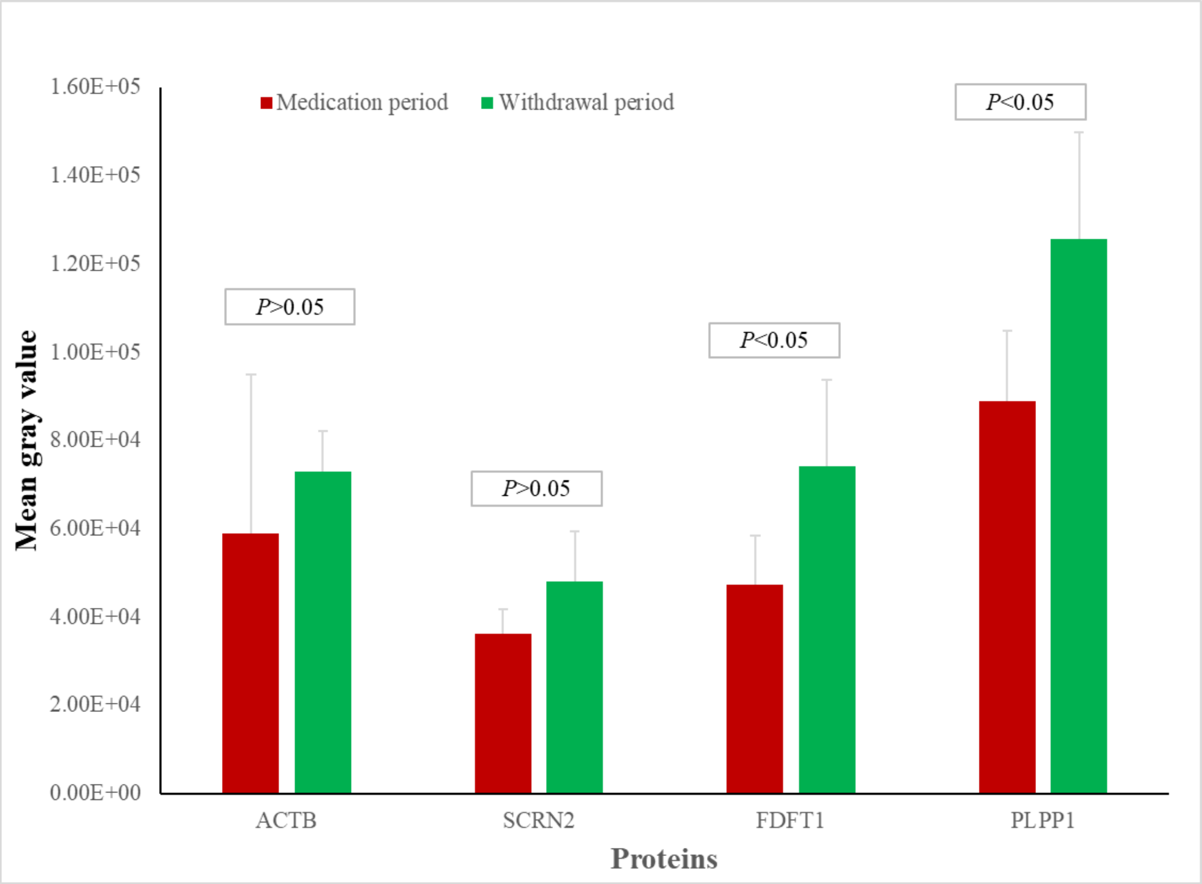

Figure S3. Statistical analysis of gray value based on western blot.

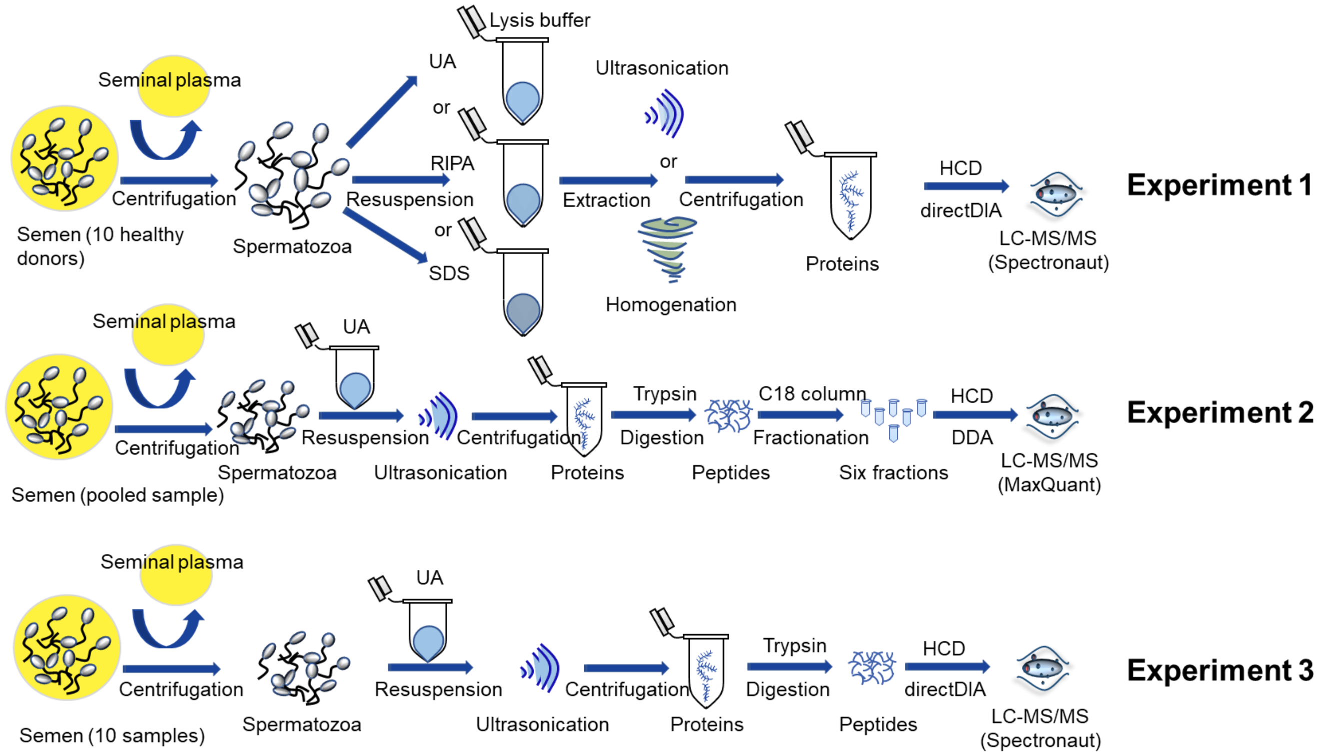

Figure S4. Overall experimental design and flow chart.
